# Preparation of Rutin–Whey Protein Pickering Emulsion and Its Effect on Zebrafish Skeletal Muscle Movement Ability

**DOI:** 10.3390/nu16183050

**Published:** 2024-09-10

**Authors:** Yiting Zhang, Wenyun Xiong, Yijing Ren, Jian Huang, Xiaoying Wang, Ou Wang, Shengbao Cai

**Affiliations:** 1 Faculty of Food Science and Engineering, Kunming University of Science and Technology, Kunming 650500, China; zyt2734803829@163.com (Y.Z.); xwy2914379541@163.com (W.X.); 15752429951@163.com (X.W.); 2 NHC Key Laboratory of Public Nutrition and Health, National Institute for Nutrition and Health, Chinese Center for Disease Control and Prevention, Beijing 100050, China; ryjing3021@163.com (Y.R.); huangjian@ninh.chinacdc.cn (J.H.); 3 School of Food and Health, Beijing Technology and Business University, Beijing 100048, China

**Keywords:** sarcopenia, nutrition prevention, bioaccessibility, molecular docking, muscle function

## Abstract

Nutritional supplementation enriched with protein and antioxidants has been demonstrated to effectively strengthen skeletal muscle function and mitigate the risk of sarcopenia. Dietary protein has also been a common carrier to establish bioactive delivery system. Therefore, in this study, a Pickering emulsion delivery system for rutin was constructed with whey protein, and its structural characteristics, bioaccessibility, and molecular interactions were investigated. In the in vivo study, zebrafish (*n* = 10 in each group), which have a high genetic homology to humans, were treated with dexamethasone to induce sarcopenia symptoms and were administered with rutin, whey protein and the Pickering emulsion, respectively, for muscle movement ability evaluation, and zebrafish treated with or without dexamethasone was used as the model and the control groups, respectively. Results showed that the Pickering emulsion was homogeneous in particle size with a rutin encapsulation rate of 71.16 ± 0.15% and loading efficiency of 44.48 ± 0.11%. Rutin in the Pickering emulsion exhibited a significantly higher bioaccessibility than the free form. The interaction forces between rutin and the two components of whey proteins (α-LA and β-LG) were mainly van der Waals forces and hydrogen bonds. After treatment for 96 h, the zebrafish in Picking emulsion groups showed a significantly increased high-speed movement time and frequency, an increased level of ATP, prolonged peripheral motor nerve length, and normalized muscular histological structure compared with those of the model group (*p* < 0.05). The results of this study developed a new strategy for rutin utilization and provide scientific evidence for sarcopenia prevention with a food-derived resource.

## 1. Introduction

With the advent of an aging society, the health of the elderly is of paramount importance. Skeletal muscle health is a non-negligible issue during aging. With an increase in age, there is a decline in skeletal muscle mass and function, which is clinically defined as sarcopenia [1,2]. It was estimated that sarcopenia impacted approximately 10% to 16% of the elderly population globally [3]. The exact prevalence rate of sarcopenia differed with the diagnostic criteria [4], but it has been well recognized that sarcopenia will increase the risk of mortality, falls, and fractures [5] and become a big threat to the elderly.

Nutritional intervention has been identified as one of the effective methods for sarcopenia prevention or treatment therapy [6]. There was a consensus that the elderly population should be advised to increase their protein intake to maintain optimal muscle function [7]. Whey protein is derived from animals and has been widely used as a nutritional supplement to achieve muscle mass or strength enhancement [8,9].

Additionally, ageing is accompanied by an imbalance in oxidative stress, which will result in damage to muscle mitochondria and membranes, and may finally lead to cell apoptosis [10,11]. Low dietary intake of antioxidants was found to be associated with lower muscle strength in the elderly [12]. A negative association between oxidative stress status and skeletal muscle mass and strength has also been shown in the US population [13]. Therefore, dietary antioxidants seem to be crucial for maintaining muscle health. Polyphenols are a kind of natural plant chemical and have been known for their outstanding antioxidant capacity. In the published studies, polyphenols such as resveratrol, luteolin, etc., could attenuate ageing-related muscle alteration [14,15]. Rutin, a flavonoid that is widely disturbed in food sources, exhibited various health benefits, like antioxidant, anti-inflammatory, hepatic protection, etc. [16,17]. A published in vivo study reported that rutin could increase the mitochondrial biogenesis in muscle [18], suggesting a potential positive effect of rutin on muscle health.

However, despite the various health benefits of rutin, its low solubility and undesirable bioavailability have limited its utilization [19]. A food delivery system is a way to overcome the problems of rutin application. It has been reported that rutin could be encapsulated in a liposome [20] or nanoparticle [21] with a food-derived carrier, and achieve improved bioavailability. A Pickering emulsion is a kind of emulsion stabilized by solid particles, and has now become a hotspot in food delivery system development [22]. Whey protein is a common ingredient for Pickering emulsion preparation [23], and can be combined with rutin in forms such as gel [24] or by crystallization [25]. However, it remains unknown whether whey protein is able to form a Pickering emulsion with rutin and exhibit health benefits on muscle.

Based on the nutrition prevention strategy for sarcopenia and the rutin utilization problems mentioned above, the aim of this study was to develop a rutin–whey protein Pickering emulsion (RWP), characterize its structural properties, and investigate its in vivo effect on muscle motor function. The results of this research may reveal the nutritional effects of the rutin–whey protein Pickering emulsion on muscle and provide scientific evidence for the prevention of sarcopenia with a food-derived resource.

## 2. Materials and Methods

### 2.1. Chemicals and Reagents

Rutin (98%) was purchased from Nanjing Jingzhu Biotechnology Co Ltd. (Nanjing, China), and whey protein (98%) was purchased from DAVISCO, USA. Edible tea oil is derived from the mature seeds of *Camellia oleifera* Abel and extracted by physical pressing; it is a natural high-grade edible vegetable oil and was purchased from Qiyunshan Foods Ltd. (Jiangxi, China). Dexamethasone was obtained from Shanghai Aladdin Biochemical Technology Co., Ltd. (Shanghai, China); an ATP assay kit was obtained from Promega, USA (Purchased from Hangzhou, China); and dimethylsulfoxide (DMSO), anhydrous ethanol, and other reagents were analytically pure.

### 2.2. Preparation of Rutin–Whey Protein Pickering Emulsion (RWP)

For the Pickering emulsion preparation, 1.6 g whey protein was dissolved in 9 mL of ddH_2_O, with the solution subjected to 50 °C in a water bath for 45 min to ensure complete dissolution. Concurrently, 200 mg rutin was dissolved in 1 mL anhydrous ethanol. Subsequently, the whey protein solution and rutin solution were combined and mixed thoroughly to achieve a homogeneous mixture. Edible tea oil was used as the oil phase, and mixed with the whey protein–rutin aqueous solution at a 1:1 volume ratio. Finally, the mixed solution was homogenized at 13,000 rpm for 2 min with an Ultraturax T18 homogeniser (IKA, Staufen, Germany) to obtain the Pickering emulsion, and the emulsion was stored at 4 °C for further analysis. In the system, the whey protein concentration was 8% wt and rutin concentration was 10 mg/mL.

### 2.3. Characterization of RWP

#### 2.3.1. Particle Size and Zeta (ζ) Potential

The particle size and ζ-potential of RWP were measured at 25 °C using a laser particle sizer, Zetasizer Nano-ZS90 (Malvern Instruments, Worcestershire, UK). The prepared Pickering emulsion was diluted to 1.0 mg/mL with deionized water and 1.0 mL was taken into a cuvette to determine the particle size. The zeta (ζ)potential was determined by pipetting an appropriate amount of the sample dilution into the potentiometric cuvette. The experiment was repeated three times.

#### 2.3.2. Confocal Laser Scanning Microscopy (CLSM) Observation

Nile Blue and Nile Red dyes were dissolved in 1,2-propanediol at a concentration of 1 mg/mL, respectively. Edible tea oil was stained with Nile Red and the whey protein solution was stained with Nile Blue. Briefly, 10 uL of each dye was thoroughly mixed with 0.5 mL of RWP. Subsequently, a 10 μL sample of the mixture was placed on a slide, covered with a coverslip and observed with CLSM. The CLSM parameters were set as follows: excitation wavelength for Nile red at 488 nm and for Nile blue at 633 nm, scan density of 1024 × 1024 pixels, and scan frequency of 100 Hz.

#### 2.3.3. Encapsulation Efficiency and Loading Capacity

Freshly prepared RWP was centrifuged at 4 °C at 12,000× *g* for 30 min. The supernatant was collected and homogeneously mixed with DMSO, and the absorbance of the solution was measured at 364 nm with a UV spectrophotometer to calculate the free rutin concentration. The encapsulation efficiency and loading capacity were calculated according to Equations (1) and (2), respectively [26]:

Encapsulation efficiency (%) = (Total rutin content − free rutin content)/(Total rutin content) × 100%(1)

Loading capacity (%) = (Load rutin quality)/(Emulsion quality) × 100%(2)

### 2.4. Storage Stability

The RWP samples were stored in the dark at 4 °C and 25 °C for 35 days, respectively, and the rutin retention was measured weekly. Briefly, 0.5 mL of emulsion was diluted in 2.0 mL organic solvent (dichloromethane–methanol = 1:1, *v*/*v*) and centrifuged at 8000× *g* for 5 min. The extraction was repeated twice and the supernatant was collected and combined for rutin concentration measurement at 364 nm. In addition, the degradation of rutin followed a first-order kinetic reaction [27], expressed as follows:

ln(c/c_0_) = −kt (3)

t_1/2_ = ln(2)/k (4)

In the equations, c and c_0_ represent the rutin concentration at a given storage time and initial time, respectively; t is the storage time (d); t_1/2_ is the half-life (d); and k is the rate constant (d^−1^ ).

### 2.5. In Vitro Digestion of Pickering Emulsion

The simulated digestion method was consistent with Ling Huang’s method with some modifications [28]. Simulated gastric fluid (SGF, 0.15 mol/L NaCl, 2000 units/mL pepsin, pH 2.0) and simulated intestinal fluid (SIF,10.0 mmol/L CaCl_2_,100 units/mL trypsin, 20.0 mg/mL bile salts, pH 7.4) were used for this in vitro digestion. For the gastric digestion, 7.5 mL RWP was mixed with 10.0 mL SGF and the pH value of the mixture was adjusted to 2.0. The system was incubated at 37 °C with continuous shaking at 100 rpm for 2 h. Then, the gastric-digested mixture was further mixed with 15.0 mL SIF, and the pH was adjusted to 7.4. The system was subsequently incubated in the dark at 37 °C with constantly shaking at 100 rpm for an additional 2 h to simulate intestinal digestion. After digestion, 4.0 mL liquid was centrifuged at 10,000× *g* for 40 min at 25 °C, and the supernatant was collected as the micellar phase. Subsequently, 1.0 mL of the micelle fraction was mixed with 2.0 mL methanol and centrifuged at 25 °C at 8000× *g* for 5.0 min. The solubilized rutin was extracted from the bottom layer, and the top layer was re-extracted with methanol following the same procedure. After extraction, the dissolved rutin in the bottom layer was collected and mixed, and the absorbance was measured at 364 nm. Retention rate and bioaccessibility of rutin were determined using Equations (5) and (6):

Retention rate (%) = M_t_/M_0_ × 100% (5)

Bioaccessibility (%) = M_micelles_/M_0_ × 100% (6)

where M_t_ is the residual content of rutin in solution, M_0_ is the initial content of rutin in fresh solution, and M_micelles_ is the content of rutin in the micellar fraction.

### 2.6. Molecular Docking

Molecular docking was performed using AutoDock Vina software (version 1.5.6) [29]. Whey proteins mainly consisted of α-lactalbumin (α-LA) and β-lactoglobulin (β-LG). The 3D structures of α-LA and β-LG were retrieved from the RCSB database (http://www.rcsb.org, accessed on 14 August 2024) (PDB IDs: 6IP9 and 3NPO) with unnecessary ligands and water molecules removed. The 3D conformation of rutin was downloaded from the PubChem database (http://pubchem.ncbi.nlm.nih.gov, accessed on 14 August 2024) (ID: 5280805). Polar hydrogen atoms and Gasteiger charges were added to α-LA and β-LG using AutoDock Tools software (ADT, version 1.5.6). To obtain the best binding conformation, the whole protein was used as a potentiometric binding site using the blind docking method. The grid coordinates used for the molecular docking studies were as follows: α-LA (x = 11.264, y = −14.727, z = −2.258) and β-LG (x = −13.099, y = 1.223, z= −1.088). Affinity and score values were used to consider the optimal docking position for rutin, and conformations and interactions were analyzed using PyMOL (version 2.3.1) (https://pymol.org/, accessed on 14 August 2024) and Discovery Studio 4.5 for visualization.

### 2.7. Zebrafish Experiment

#### 2.7.1. Experimental Animals

Wild-type AB strain zebrafish bred in natural pairs and aged 3 days post-fertilization (3 dpf) were used for a maximum test dose (MTD) assay, ATP content test, mobility ability evaluation, and skeletal muscle histopathological analysis. Tg(NBT:MAPT-GFP) zebrafish (NBT strain) aged 3 dpf were used for a peripheral motor nerve length assay. For the MTD assay, 450 zebrafish were used, and for the parameter measurements, 360 zebrafish were used. Therefore, a total of 810 zebrafish were used in this study.

All the zebrafish were raised in fish culture water at 28 °C. The breeding service was provided by Hunter Biotechnology, Inc (Hangzhou, China), [30,31]. The license for the use of experimental animals was No. SYXK (Zhe) 2022–0004, the feeding management was in accordance with the requirements of the international AAALAC certification (certification No. 001458), and the IACUC ethical approval number was IACUC-2023-5617-01 (approval date: 17 May 2023).

#### 2.7.2. Maximum Tolerated Dose Assay

Wild-type AB strain zebrafish were distributed in six-well microplates, with 30 zebrafish per well in 3 mL fish water for each treatment. Rutin was dissolved in 50% ethanol, and whey protein and RWP were dissolved or diluted with standard dilution water. Samples with different concentrations were given to the zebrafish by enteral injection. The mortality of the zebrafish was observed daily and the culture environment was refreshed every 48 h. After 96 h of treatment, the maximum tolerated dose (MTD) was calculated.

#### 2.7.3. Mobility Ability Evaluation

Wild-type AB strain zebrafish were cultured as described above and grouped as control (C), model (M), positive control (P, trimetazidine hydrochloride), rutin (R), whey protein (WP), and RWP. Samples were given to the zebrafish by enteral injection, and the concentration was determined based on the MTD assay. The symptoms of sarcopenia in zebrafish were induced with dexamethasone aqueous solution, except in the C group. After administration of the different treatments for 96 h, ten of the wild-type AB strain zebrafish from each group were distributed in a 96-wells microplate, with one fish per well in 200 μL fish water for movement observation. The movement of zebrafish in one hour was tracked and analyzed with ViewPoint behavior system (Zebra Lab 3.11, ViewPoint, France). Briefly, the movement of zebrafish was divided into three categories according to the speed: low-speed movement (less than 4 mm/s), medium-speed movement (4–20 mm/s), and high-speed movement (higher than 20 mm/s). The movement routes of the zebrafish were automatically tracked and the time duration and frequency of high-speed movements were recorded.

#### 2.7.4. ATP Level Test

Wild-type AB strain zebrafish were grouped and treated as described above. After treatment for 96 h, ten zebrafish from each group were collected and the ATP contents were assayed with a commercial kit.

#### 2.7.5. Peripheral Motor Nerve Length Measurement

The Tg(NBT:MAPT-GFP) zebrafish were grouped and treated as the wild-type AB strain zebrafish above. After 96 h treatment, ten NBT zebrafish from each group were selected for fluorescence microscope photography. The length of zebrafish peripheral motor nerves was analyzed with NIS-Elements D 3.20 image processing software.

#### 2.7.6. Histopathological Analysis

Ten wild-type AB strain zebrafish from different groups were fixed, dehydrated, embedded, sectioned, and stained with hematoxylin and eosin as described in published reports [32,33]. The histological structure of skeletal muscle was observed microscopically.

### 2.8. Statistical Analysis

For emulsion preparation and characterization, all experiments were repeated three times and there were three parallels for each test. Data were expressed as mean ± standard deviation (SD) and analyzed with SPSS 20.0. In the zebrafish experiment, results were expressed as mean ± standard errors (SE) and analyzed with SPSS 22.0. A Kolmogorov–Smirnov test was conducted to determine whether data were normally distributed. For normally distributed data, an independent t-test was conducted; otherwise, a Mann-Whitney U test was used. The statistical significance was set when the *p* value was less than 0.05.

## 3. Results and Discussion

### 3.1. Structural Characteristics and Loading Efficiency of Emulsions

**Figure 1:**
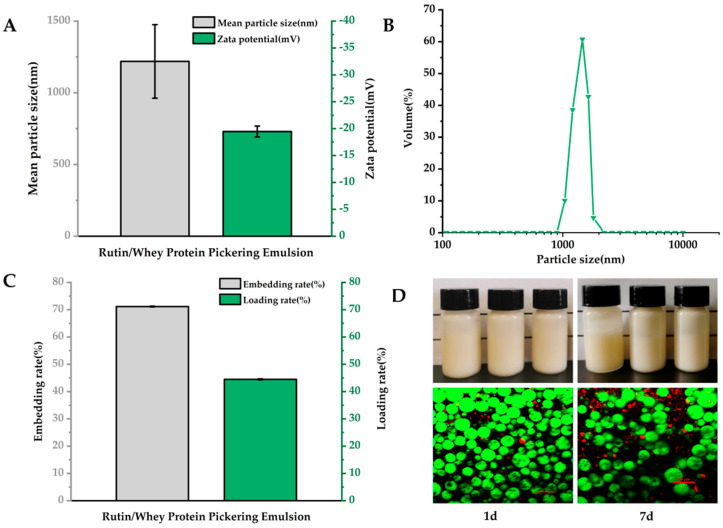
Characterization of rutin–whey protein Pickering emulsion (RWP). (**A**) Particle size and zeta potential. (**B**) Particle size distribution. (**C**) Embedding rate and loading rate. (**D**) Apparent images of fresh emulsions and emulsions stored for seven days (top) and confocal laser scanning microscopy images (bottom). Data are expressed as mean ± SD (*n* = 3).

As data shown in Figure 1A, the average particle size of RWP was 1219 ± 256.36 nm, and the zeta potential was −19.47 ± 1.01 mV, which was similar with previous results [34]. The RWP particle size distribution was unimodal, indicating a homogeneous emulsion particle size (Figure 1B). Rutin has previously been delivered with glucose glycosylated zein with an encapsulation efficiency of 62.43% [35]. However, in the current study, the encapsulation rate of rutin in RWP was 71.16 ± 0.15%, and the loading rate was 44.48 ± 0.11% (Figure 1C), indicating that RWP showed good embedding properties.

The structural properties of RWP were observed by CLSM. The staining of the oil phase and protein revealed that the oil phase (in a red color) was inside the droplets in the freshly prepared emulsions, whereas the whey protein (in a green color) formed a dense stacked layer at the droplet boundaries (Figure 1D, bottom), which provided a dense barrier for the oil droplets to prevent agglomeration and Ostwald maturation, and this result shows that the RWP was an oil-in-water emulsion [36]. The close stacking of the emulsion droplets allows them to interact strongly with each other, which helps to increase the strength and stability of the gel network [37]. When stored for seven days, the oil droplets became larger and less dense, but there was no emulsion breakage or emulsion precipitation (Figure 1D, bottom), which was consistent with the image observation (Figure 1D top), indicating good emulsion stability.

### 3.2. Storage Stability

**Figure 2:**
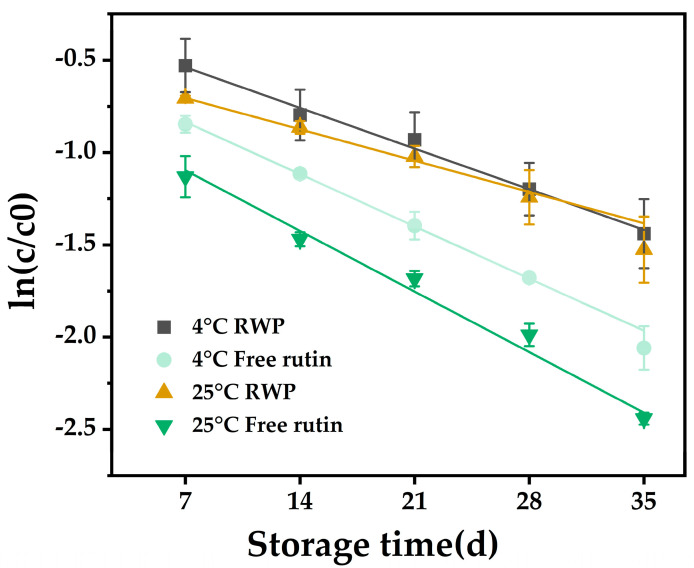
The first-order kinetics of RWP and rutin solution stored at 4 °C and 25 °C for 35 days. RWP: rutin–whey protein Pickering emulsion. Data are expressed as mean ± SD (*n* = 3).

The study of degradation kinetics is important for predicting the stability of bioactives during storage and processing [38]. Figure 2 shows the degradation curves of free and encapsulated rutin stored at 4 °C and 25 °C for 35 days, respectively, and all the curves showed good linear correlation coefficients (R^2^ > 0.97). This indicated that the model fitted the degradation kinetics well [39].

The results in Table 1 show that, whether stored at 4 °C or 25 °C, the k-values of encapsulated rutin were lower and the t_1/2_ values were higher when compared with those of free rutin, indicating that the degradation rate of rutin was slowed down after encapsulation. Moreover, the k-value of RWP at 4 °C (0.0411) was lower than that at 25 °C (0.0436). These results suggested that loading of rutin with whey protein may improve its storage stability, and it may be more favorable for emulsion storage at low temperatures.

Literature studies have shown that the stability of bioactives in emulsion systems are influenced by the structure of the emulsion system [40]. Emulsion gels with higher mechanical properties (e.g., hardness or storage modulus) are better able to slow down lipid oxidation and degradation of the contained bioactives [41]. Pickering emulsions, as a type of emulsion gel, may provide physical protection to the encapsulated rutin [39]. In addition, strong interactions between the interface and the gel matrix also favor the stability of the bioactives [42]. Thus, the encapsulated rutin in RWP may be protected by the whey protein interactions and the thick layer formed by emulsion droplets on the surface.

### 3.3. In Vitro Simulation of Digestion and Bioaccessibility

**Figure 3:**
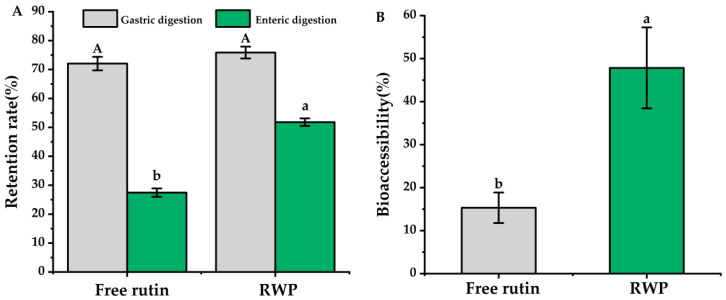
In vitro digestion simulation. (**A**) Retention rate. (**B**) Bioaccessibility. RWP: rutin–whey protein Pickering emulsion. Data are expressed as mean ± SD (*n* = 3). Different letters indicate significant differences (*p* < 0.05). Uppercase letters in (**A**) indicate significant differences in gastric digestion and lowercase letters indicate significant differences in enteric digestion.

It has been reported that some flavonoids, represented by rutin, are susceptible to isomerization and protonation under acidic conditions, leading to degradation and reduced bioavailability [43]. Therefore, the gastrointestinal digestive stability of rutin was investigated in this study. As the results in Figure 3 show, after gastric and enteric digestion, the retention rate of free rutin was 27.45 ± 1.45%, while the retention rate of encapsulated rutin was over 50%, which showed significant difference (*p* < 0.05). Additionally, the bioaccessibility of rutin in RWP was 47.84 ± 9.43%, which was about three times higher than that of the free rutin (*p* < 0.05).

The results in Figure 3 suggest that the edible tea oil and whey protein system could improve the stability and bioaccessibility of the encapsulated rutin. In the emulsion system, rutin could be encapsulated in the hydrophobic core of the edible tea oil–whey protein system through non-covalent interactions, including hydrogen bonding and electrostatic, and hydrophobic interactions, and may form a spatial site barrier to prevent pepsin digestion [44]. Furthermore, from the image shown in Figure 1D, whey protein forms a thick layer on the surface of the emulsion droplets, which may prevent the digestion and degradation of rutin in the acidic gastric environment.

The release and solubilization of fat-soluble nutrients mainly occurs during small intestinal digestion, which depends on a variety of exogenous factors such as molecular properties, food matrix, processing, and interfacial composition [45]. The transport of lipophilic substances between different colloidal phases is also controlled by a number of determinants, such as the lipophilicity of the substance and the affinity of the various lysophilic phases [46]. In the current study, the monounsaturated fatty acids in edible tea oil are hydrophobic, which may lead to a great ability to dissolve and transport rutin, thus improving its bioaccessibility [44].

### 3.4. Molecular Docking

**Figure 4:**
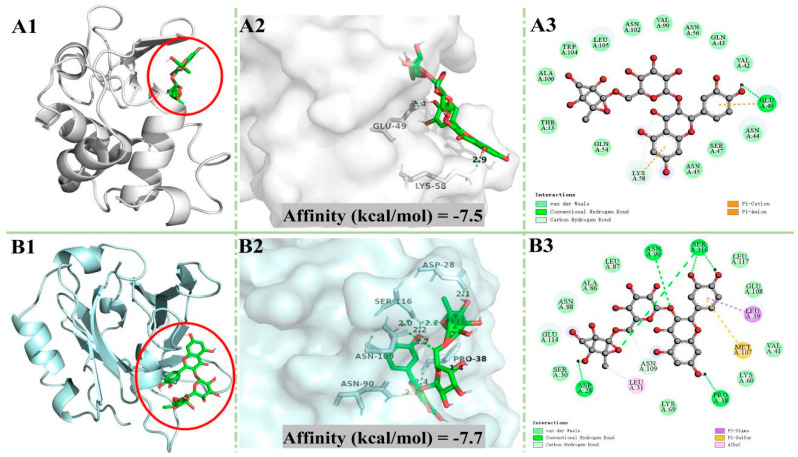
Molecular docking results. (**A**) α-LA + rutin. (**B**) β-LG + rutin. α-LA: α-lactalbumin; β-LG: β-lactoglobulin; panel labels 1, 2, and 3 represent the 3D binding site map, 3D hydrogen bonding map, and 2D interaction map, respectively.

In order to better understand the interaction mechanism of rutin with whey protein, molecular docking analysis was conducted. α-LA and β-LG were the most prominent proteins in whey. The optimal docking conformation between rutin and the two proteins are shown in Figure 4. In general, the internal cavities of proteins can provide the ligand with a strong hydrophobic environment and multiple hydrogen bonding sites, which contribute to the stability of the ligand [47]. It can be seen in Figure 4 that rutin could bind to the inner cavities of α-LA and β-LG to form a relatively stable conformation. As the results in Table 2 show, rutin forms one hydrogen bond and 16 non-hydrogen bonds with α-LA, and 13 of the non-hydrogen bonds were van der Waals forces, while it formed six hydrogen bonds and 14 non-hydrogen bonds with β-LG, and 10 of the non-hydrogen bonds were van der Waals forces. Such results indicated that rutin could bind to α-LA and β-LG to from a stable conformation mainly through van der Waals forces and hydrogen bonds [48].

The absolute value of the binding affinity denotes the potential interaction strength between ligand and protein. In molecular docking studies, the hydrogen bond is the main force that maintains the stability of small molecules and protein complexes. As shown by the data Table 2, the absolute affinity value and the number of hydrogen bonds of β-LG + rutin were higher than those of α-LA + rutin, suggesting that β-LG may be the main carrier of rutin in RWP.

### 3.5. Effects of the RWP on Zebrafish Muscle Motor Function

The zebrafish has become a reliable animal model for biochemical studies. Its genome shows high similarity to that of humans, with about 70% of genes being analogous to humans [49]. In addition, zebrafish muscle also shares a high similarity to human skeletal muscle, making it a suitable model for sarcopenia research [50,51,52]. In the current study, rutin, whey protein, and their Pickering emulsion were applied to zebrafish to observe their effects on the muscle motor function.

The results of the MTD assay revealed the safety of the different doses to zebrafish. In this study, five concentrations of each treatment were applied to zebrafish, and the results in Table 3 show that a concentration of whey protein up to 2000 ng/fish was safe for zebrafish survival. The maximum safe dose for rutin was 50.0 ng/fish, and 50% RWP was safe for zebrafish.

Based on the results of the MTD assay and the design of the Pickering emulsion, two concentrations were selected for the following tests in each group. In the R group, rutin was applied at 25.0 ng/fish (R1) and 50.0 ng/fish (R2), respectively; in the W group, whey protein was applied at 200.0 ng/fish (W1) and 400.0 ng/fish (W2), respectively; in the RWP group, the Pickering emulsion was applied at 25% (RWP1) and 50% (RWP2) of the initial concentration, respectively. Concentrations of rutin and whey protein at both doses remained consistent with those in the Pickering emulsion groups.

**Figure 5:**
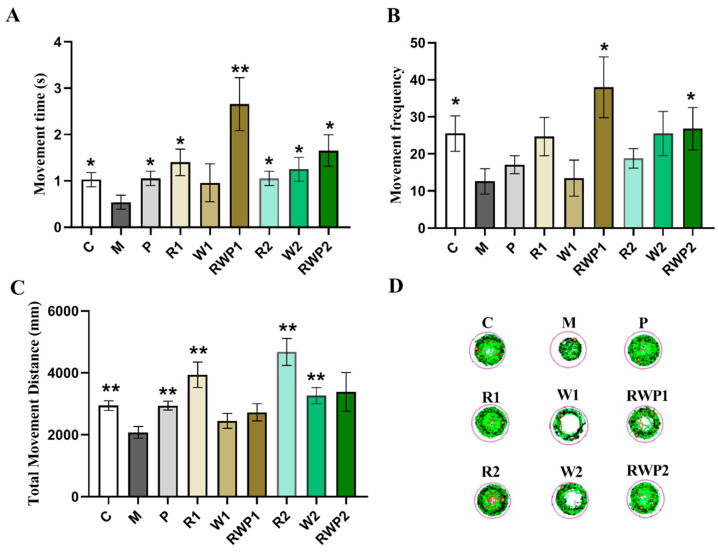
Movement performance of zebrafish. (**A**) High-speed movement time. (**B**) High-speed movement frequency. (**C**) Total movement distance. (**D**) Trace map of the movement. A moving speed lower than 4 mm/s is marked in green; 4–20 mm/s is marked in black; and higher than 20 mm/s is marked in red. C, the control group; M, the model group; P, the positive control group; R1 and R2, the rutin treatments at 25.0 ng/fish and 50.0 ng/fish, respectively; W1 and W2, the whey protein treatments at 200.0 ng/fish and 400.0 ng/fish, respectively; RWP1 and RWP2, the rutin–whey protein Pickering emulsion treatments at 25% and 50% of the initial concentration, respectively. Data are expressed as mean ± SE (*n* = 10). The result of each group was compared with the M group, * indicates *p* < 0.05 and ** indicates *p* < 0.01.

A behavior test of zebrafish can directly reflect their motor activity, which may reveal the function of the skeletal muscle. As results show in Figure 5, zebrafish in the M group exhibited shorter movement time and distance, less movement frequency, and the trace map exhibited fewer lines, indicating a potential decline in muscle motor function (compared with the C group, *p* < 0.05).

Flavonoids are not only natural antioxidants but also potential agents for muscle protection [53]. As a kind of typical flavonoid, rutin treatments at both of the two administered concentrations significantly elevated the high-speed movement time and distance of the zebrafish (in comparison with the M group, *p* < 0.05 for movement time, *p* < 0.01 for movement distance). Consistently, much more condensed motor lines were traced in Figure 5D. This result was in accordance with a previous report that the rutin-enriched extract could enhance swimming endurance in rats [54], demonstrating the potential ability of rutin to improve muscle locomotion.

Whey protein is beneficial for skeletal muscle health, stimulating protein synthesis and attenuating muscle loss [55]. In exercising mice, whey protein administration could significantly prolong the endurance time and increase grip strength [56]. In this study, when compared with that of the M group, a high level of whey protein (W2) could significantly elevate the movement distance by 57.1% (*p* < 0.01), and its high-speed movement time was about 2.3 times that of the M group (*p* < 0.05).

In addition to the health benefits on muscle, whey protein also exhibits an important function in the food delivery system of bioactive compounds. It has been reported that whey protein could be used as a carrier to deliver bioactive compounds such as soy isoflavones, EGCG, etc., with enhanced bioavailability, stability, and transcellular transport [57,58]. When whey protein was combined with rutin as a Pickering emulsion, it could significantly increase the high-speed movement time and high-speed movement frequency of the zebrafish at both low and high doses (compared with the M group, *p* < 0.05). As shown in Figure 5A,B, the high-speed movement time of RWP1 was about 4.9 times that of M (*p* < 0.01), while the high-speed movement frequency was about 3.0 times (*p* < 0.05).

Among the three treatments, RWP was the only treatment that exhibited a significant influence on the high-speed movement frequency, but it showed an insignificant effect on the total movement distance of the zebrafish (Figure 5C). The results suggested that the effect of a rutin and whey protein combined Pickering emulsion on skeletal muscle may not be consistent with their individual effects when administered separately. The RWP might be more pronounced in eliciting rapid movements rather than affecting the entire spectrum of locomotive behavior.

**Figure 6:**
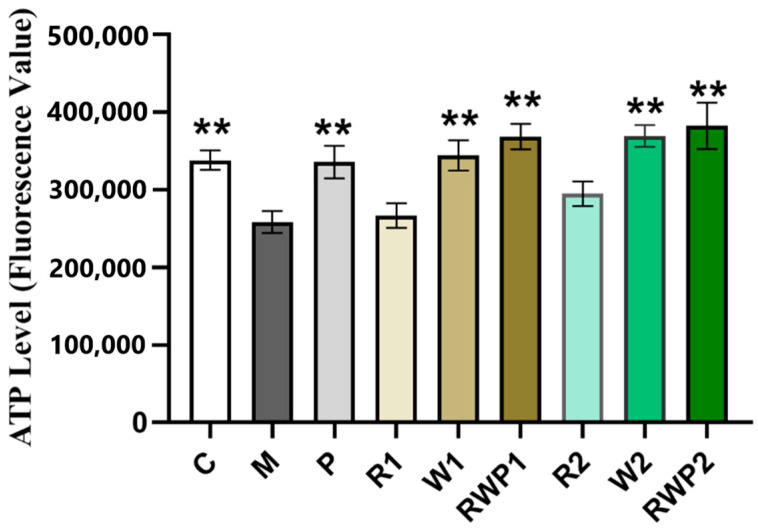
ATP levels in different groups. C, the control group; M, the model group; P, the positive control group; R1 and R2, the rutin treatments at 25.0 ng/fish and 50.0 ng/fish, respectively; W1 and W2, the whey protein treatments at 200.0 ng/fish and 400.0 ng/fish, respectively; RWP1 and RWP2, the rutin–whey protein Pickering emulsion treatments at 25% and 50% of the initial concentration, respectively. Data are expressed as mean ± SE (*n* = 10). The result of each group is compared with the M group, * indicates *p* < 0.05 and ** indicates *p* < 0.01.

During exercise, muscle contraction requires a large amount of energy, which is mainly derived from ATP [59]. As shown in Figure 6, the ATP levels in the W1 and W2 groups were both significantly higher than those in the M group (*p* < 0.01), while rutin treatment had an insignificant effect (*p* > 0.05). A previous report proved that rutin could increase the mitochondrial size and gene expression levels associated with mitochondrial biogenesis, but whether it affected the mitochondrial ATP content remained unknown [18]. After treatments RWP1 and RWP2, the ATP levels in the zebrafish were remarkably elevated by 42.7% and 48.1%, respectively (compared with the M group, *p* < 0.01). The elevated ATP levels in the RWP groups would provide more energy to the zebrafish skeletal muscle and may account for the significant enhancing effect on the time and frequency of high-speed movement.

**Figure 7:**
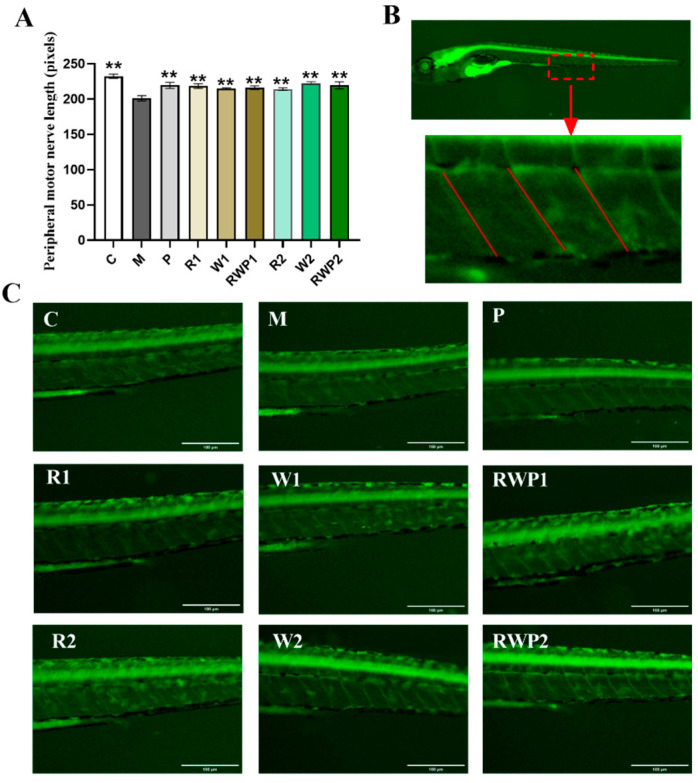
The peripheral motor nerve length measurement results and images (120×). (**A**) Peripheral motor nerve length. (**B**) Schematic representation of the peripheral motor nerve length analysis region. (**C**) Peripheral motor nerve length measurement areas of each treatment. C, the control group; M, the model group; P, the positive control group; R1 and R2, the rutin treatments at 25.0 ng/fish and 50.0 ng/fish, respectively; W1 and W2, the whey protein treatments at 200.0 ng/fish and 400.0 ng/fish, respectively; RWP1 and RWP2, the rutin–whey protein Pickering emulsion treatments at 25% and 50% of the initial concentration, respectively. Data are expressed as mean ± SE (*n* = 10). The result of each group was compared with the M group, * indicates *p* < 0.05 and ** indicates *p* < 0.01.

Peripheral motor nerves play an important role in maintaining normal skeletal muscle movement and function. Damage or dysfunction of the peripheral motor nerves can result in muscle weakness, paralysis, or other movement disorders [60,61]. The Tg(NBT:MAPT-GFP) zebrafish is a transgenic strain that expresses green fluorescent protein in the peripheral motor neurons [62]. In the present study, the peripheral motor nerve length of the zebrafish was measured, and the results are shown in Figure 7. Compared with the result of the M group, all the interventions could significantly elevate the length of the peripheral motor nerves (*p* < 0.05).

**Figure 8:**
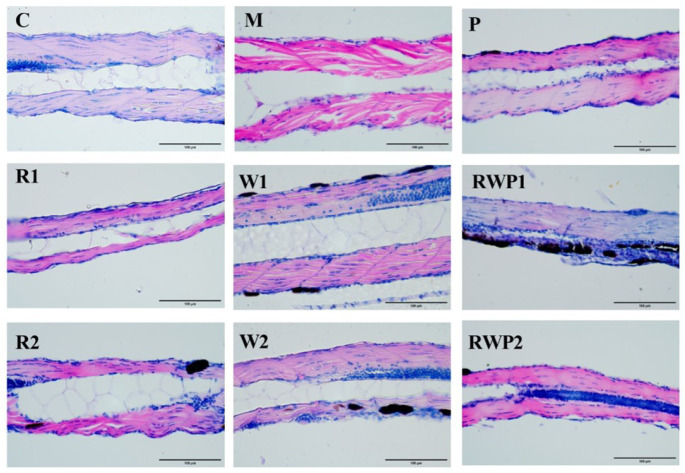
The histopathological analysis of zebrafish skeletal muscle (400×). C, the control group; M, the model group; P, the positive control group; R1 and R2, the rutin treatments at 25.0 ng/fish and 50.0 ng/fish, respectively; W1 and W2, the whey protein treatments at 200.0 ng/fish and 400.0 ng/fish, respectively; RWP1 and RWP2, the rutin–whey protein Pickering emulsion treatments at 25% and 50% of the initial concentration, respectively.

Aging is accompanied by damage to skeletal muscle structure, which is also one of the typical symptoms of sarcopenia [63]. In the histopathological analysis (Figure 8), the muscle tissue of the C group zebrafish exhibited a typical organization, with muscle cells being slender and elongated, showing a fibrous appearance, and tightly arranged. In contrast, the muscle tissue of the M group was loosely structured and the myocytes were irregularly arranged, indicating the compromised muscle structure. The zebrafish muscle tissue lesions of the R, W, and RWP groups at various doses showed a normalization tendency when compared to the M group. The arrangement of myocytes in these groups was more regular and tighter than those of the M group, indicating the protective effect on skeletal muscle structure.

As previously reported, rutin could prevent dexamethasone-induced muscle loss through blocking the protein degradation pathway [64]. Its antioxidant ability may also help to eliminate aging-related oxidative stress and be beneficial in maintaining normal muscle structure and function [65]. Additionally, whey protein is rich in branched-chain amino acids, and has been widely proved to be an effective food component for muscle protein synthesis and function maintenance [66,67]. In this study, treatments of rutin, whey protein, and RWP all exhibited significant improvement effects on zebrafish muscle motor function. However, considering the increased bioaccessibility of rutin in RWP, the intervention effect of RWP may not be the simple summation of the individual impacts of free rutin and whey protein.

## 4. Conclusions

In this study, a Pickering emulsion delivery system for rutin was constructed with whey protein. The emulsion showed uniform particle size, high stability, and efficient encapsulation and loading of rutin. The bioaccessibility of rutin in the emulsion was also significantly increased. Molecular docking revealed that rutin could bind to whey proteins mainly through van der Waals forces and hydrogen bonds. In addition, the results of the in vivo experiments indicated that the rutin–whey protein emulsion could significantly promote skeletal muscle movement function in zebrafish. The results of this study developed a new strategy for the use of rutin and provide scientific evidence for the prevention of sarcopenia with a food-derived resource. In future, the in vivo effects and underlying mechanisms of RWP on muscle health need to be further explored in mammals.

## Figures and Tables

**Table 1:** The degradation kinetic parameters of RWP and rutin during storage.

Storage Temperatures	Specimens	K (d^−1^)	t_1/2_ (d)	R^2^
4 °C	RWP	0.0411	16.86	0.9931
	Free rutin	0.0588	11.79	0.9979
25 °C	RWP	0.0436	15.90	0.9816
	Free rutin	0.0698	9.93	0.9733

RWP: rutin–whey protein Pickering emulsion; K: rate constant (d^−^^1^); t_1/2_: half-life (d); R^2^: correlation coefficient.

**Table 2:** Molecule docking results of rutin with α-lactalbumin (α-LA) and β-lactoglobulin (β-LG).

Complexes	Affinity (kcal/mol)	Number of van der Waals Forces	Amino Acid Residues Involved in van der Waals Forces	Number of Hydrogen Bonds	Amino Acid Residues Involved in Hydrogen Bonds
α-LA + Rutin	−7.5	13	GLN (54,43), THR33, ALA106, TRP104, LEU105, ASN (102,45,56,44) VAL (99,42) SER47	1	GLU49(2.4 Å)
β-LG + Rutin	−7.7	10	SER30, GLU (114,108), ASN88, ALA86, LEU (87,117), VAL41, LYS (60,69)	6	SER116 (2.0 Å, 2.2 Å, 2.2 Å), ASN90 (2.3 Å), PRO38 (2.4 Å), ASP28 (2.1 Å)

GLN: glutamine; THR: threonine; ALA: alanine; TRP: tryptophan; LEU: leucine; ASN: asparagine; VAL: valine; SER: serine; GLU: glutamic acid; LYS: lysine; PRO: proline; ASP: aspartic acid.

**Table 3:** Results of MTD assay.

Groups ^1^	Concentrations	Number of Deaths	Mortality Rate (%)
Rutin	6.25 ng/fish	0	0
	12.5 ng/fish	0	0
	25.0 ng/fish	0	0
	50.0 ng/fish	0	0
	100.0 ng/fish	1	3.33
Whey protein	125.0 ng/fish	0	0
	250.0 ng/fish	0	0
	500.0 ng/fish	0	0
	1000.0 ng/fish	0	0
	2000.0 ng/fish	0	0
Pickering emulsion	6.25%	0	0
	12.5%	0	0
	25.0%	0	0
	50%	0	0
	100%	5	16.67

^1^ Samples were diluted with water, and the administration volume was 10 nL. *n* = 30 for each treatment.

## Data Availability

The original contributions presented in the study are included in the article, further inquiries can be directed to the corresponding author.
